# Association between acute aortic dissection and the distribution of aortic calcification

**DOI:** 10.1371/journal.pone.0219461

**Published:** 2019-07-11

**Authors:** Chih-Jen Yang, Shih-Hung Tsai, Jen-Chun Wang, Wei-Chou Chang, Chih-Yuan Lin, Zun-Cheng Tang, Hsian-He Hsu

**Affiliations:** 1 Department of Emergency Medicine, Tri-Service General Hospital, National Defense Medical Center, Taipei, Taiwan; 2 Department of Physiology and Biophysics, Graduate Institute of Physiology, National Defense Medical Center, Taipei, Taiwan; 3 Department of Radiology, Tri-Service General Hospital, National Defense Medical Center, Taipei, Taiwan; 4 Division of Cardiovascular surgery, Department of Surgery, Tri-Service General Hospital, National Defense Medical Center, Taipei, Taiwan; 5 Department of Biological Imaging and Radiological Science, National Yang-Ming University, Taipei, Taiwan; NIHR Leicester Biomedical Research Centre, UNITED KINGDOM

## Abstract

**Objective:**

Aortic calcification (AC) is associated with increased risks of cardiovascular events and mortality. Numerous studies have explored the association between calcification and abdominal artery aneurysm. However, evidence regarding the association between AC and acute aortic dissection (AAD) is limited. We aimed to evaluate the association between AC-related variables and the development of intimal tear (IT) in patients with AAD.

**Methods:**

We conducted a retrospective observational study involving 64 patients with type A AAD and 32 patients with type B AAD from February, 2011 to January, 2017 at a tertiary referral medical center in Taiwan. We used the default analysis module “calcification score analysis” to calculate all the calcification variables, including AC scores and volume.

**Results:**

We identified an association between AC and AAD. Patients with AAD had a greater AC volume in the aortic arch and greater AC scores for both the ascending aorta and the aortic arch than did patients without AAD. However, hypertension and coronary artery disease, rather than AC remained to be the independent risk factor for AAD in multivariate analysis. Patients with type A AAD had greater mean and cumulative AC volumes in the aortic arch, greater cumulative AC volumes in the whole aorta and higher cumulative AC scores in the aortic arch than did patients with type B AAD. ACs were superimposed on ITs in nearly half of the patients with AAD. In patients with type A AAD, AC was more commonly located distal to the IT and farther from the IT.

**Conclusions:**

We identified the associations between AC-related variables and the location of IT in patients with AAD. However, AC was not an independent risk factor for AAD. The distribution of AC was different between patients with type A and type B AAD.

## Introduction

Aging, dyslipidemia, tobacco use, inflammatory disease, chronic kidney disease and diabetes mellitus can predispose individuals to aortic calcification (AC). Calcification is a major feature of atherosclerotic cardiovascular disease. Acute aortic syndrome represents a group of potentially lethal aortic diseases and includes classic acute aortic dissection (AAD), intramural hematomas, and penetrating atherosclerotic aortic ulcers.[1] Improvements in the diagnosis and treatment of acute aortic syndrome have not resulted in a significant reduction in hospital mortality.[2] Elastic lamina degradation and AC are common features of aortic pathologies such as abdominal aortic aneurysms (AAAs) and AAD.[3] Traditional cardiovascular risk factors are related to both the incidence and progression of AC.[4] AC is associated with increased risks of cardiovascular events, stroke, coronary artery disease (CAD), cardiovascular mortality and all-cause mortality.[5] Compared with patients undergoing elective AAA repair, patients with symptomatic or ruptured AAAs exhibit an increased degree of calcification.[6] The presence of AC increases the peak wall shear stress (WSS) and decreases the biomechanical stability of AAAs.[7] AC may be induced and accelerated by elastin degradation. In animal studies, AC can be induced by genetic hyperlipidemia and nicotine.[8, 9] Initial intimal tear (IT) locations usually coincide with regions of maximal pressure or WSS.[10] The process of calcification and WSS activate several molecular mechanisms that predispose individuals to ITs. We hypothesized that the distribution and extent of AC may change the biomechanical properties of the aorta. Several studies have investigated the association between AC and AAA; however, few studies have evaluated the association between AC and AAD.[11] We aimed to evaluate the associations between AC-related variables and the development of ITs in patients with AAD.

## Methods

### Patients

We conducted a retrospective observational study of consecutive patients admitted to the emergency department with suspected AAD between February 1, 2011, and January 31, 2017. The institutional review board for human investigations of a tertiary referral medical center (Tri-Service General Hospital) approved this study and waived the requirement for informed consent because the medical records were de-identified and the study was retrospective in nature. The methods were carried out in accordance with approved guidelines. Patients with AAD were identified using electronic medical records. We omitted 11 patients without complete data. Patients who were younger than 18 years old, had not undergone chest computed tomography angiography (CTA), were diagnosed with traumatic AAD, intramural hematomas, recognised aortopathies (such as confirmed AAD before enrollment, Mönckeberg’s sclerosis, Takayasu’s arteritis, and bicuspid aortic valve disease) were excluded. For comparison, a group of control candidates was selected from individuals who had CTA exams performed in the emergency department for suspected AAD but had negative results. Patients in the study and control groups were selected by 1:1 matching by age and sex. We retrospectively reviewed the medical records of patients to collect their demographic data, including age, gender and history of smoking, and comorbidities, including hypertension (diagnosed before emergency department admission), diabetes, dyslipidemia, CAD, chronic kidney disease and previous cardiac surgery.

### Measurement of calcifications on CTA

**Fig 1:**
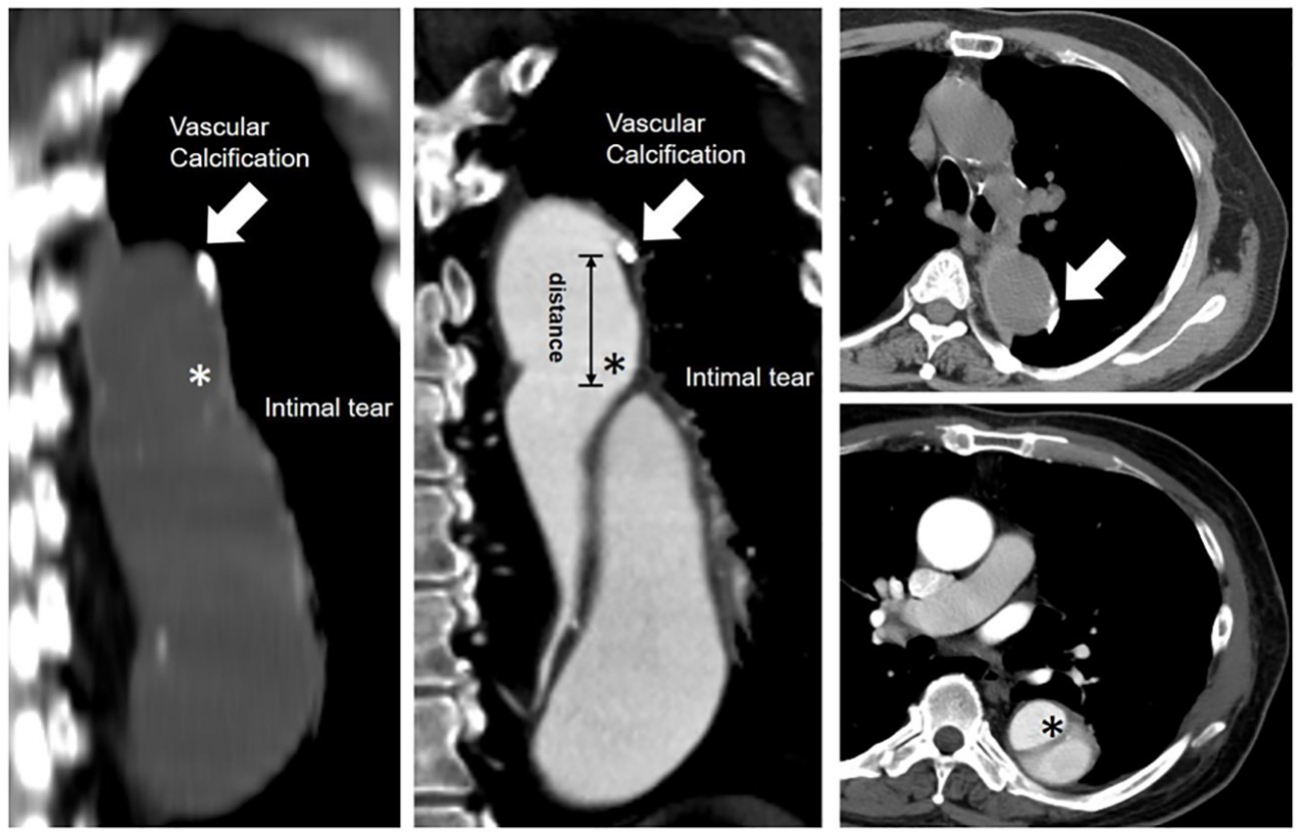
Representative figure of the determination of variables associated with aortic calcification (arrows) and intimal tears (asterisks).

We acquired the source files of these patients from a picture archiving and communication system (PACS) and transferred the data to a Philips workstation (Philips Brilliance iCT, Philips Healthcare, Best, The Netherlands). We used the default analysis module “calcification score analysis” on the Philips workstation. AC was defined that density above 130 Hounsfield unit on CT and meet the criteria as follow: (1) inside the aortic vessel. (2) consecutively show in more than 2 transverse-view on CT images. Initial IT was defined as the proximal end of the intimal flap. All the calcification variables, including AC scores and volume, were calculated. The different anatomical portions of the aorta were divided into the ascending aorta, aortic arch and descending aorta. The ascending aorta extends from the root to the origin of the right brachiocephalic artery; the arch, from the right brachiocephalic artery to the attachment of the ligamentum arteriosum; and the descending aorta, from the ligamentum arteriosum to the aortic hiatus in the diaphragm as described in the CT angiography.[12] The locations of AC were further classified according to the Ishimaru zone classification.[13] The distance between the IT and the nearest calcified plaque was also measured, as illustrated in Fig 1. A positive value indicated that the nearest calcified plaque was distal to the IT, whereas a negative value indicated that the nearest calcified plaque was proximal to the IT.

The calcification-related variables were defined as follows:

CumulativeACvolume=∑Calcifiedplaqueswithintheaorta

$$\text{Mean}\;\text{AC}\;\text{volume}=\frac{\sum Calcified\;area\;within\;aorta}{Number\;of\;calcified\;plaques}$$

$$\text{Cumulative}\;\text{AC}\;\text{scores}=\sum_{1}^{n}AC\;scores\;within\;the\;aorta\;$$

$$\text{Mean}\;\text{AC}\;\text{scores}=\frac{\sum_{1}^{n}AC\;scores\;}{Number\;of\;calcified\;plaques}$$

AC score was defined as Hounsfield units on CT multiplied by volume (mm^3^).

### Statistical analysis

Continuous data are expressed as the mean ± standard deviation and analyzed using a two-tailed Student’s *t*-test. Categorical data are expressed as frequencies (%) and evaluated using the chi-square test or Fisher’s exact test. Univariate and multivariate logistic regression analyses were performed to identify the risk factors associated with AAD. Variables of interest and those associated with p < 0.05 in the univariate analysis were entered into the multivariate logistic regression analysis. We also performed observer variability assessment for quality control by calculating intraclass correlation coefficient and Kappa value. The data were analyzed using Statistical Package for the Social Sciences version 22.0 statistical software (SPSS, Inc., Chicago, IL, USA).

## Results

### Demographic data of the patients

Overall, 97 patients who had been diagnosed with AAD during the study period were included in the analysis. One patient was excluded as an outlier since the location of the AC deviated markedly from the locations of ACs in the other patients (deviation score >3). Sixty-four patients had type A AAD and 32 patients had type B AAD. Table 1 shows the characteristics of the enrolled patients. An additional 96 age- and gender-matched patients who were admitted to the emergency department and had chest CTA performed during the same period but did not have AAD were designated as the control group. Compared with patients without AAD, patients with AAD had significantly higher rates of hypertension and greater cumulative AC volumes in the aortic arch and cumulative AC scores for both the ascending aorta and the aortic arch. For the details of surgery, 3 of 4 with aortic dissection received coronary artery bypass graft surgery, and the remaining one received Bentall procedure. 2 of 5 without aortic dissection received mitral valve replacement, and the remaining three received coronary artery bypass graft surgery. As shown in Table 2, a multivariable regression analysis revealed that hypertension (Hazard Ratio (HR) = 9.386, 95% confidence Interval (CI) = 4.883–19.654, *p* < 0.001) and CAD (HR = 3.486, 95% CI = 1.340–9.068, *p* = 0.010) were the major determinants for the development of AAD. AC volume (HR = 1.009, 95% CI = 0.987–1.032, *p* = 0.428) and scores (HR = 1.015, 95% CI = 0.993–1.037, *p* = 0.196) were not independent risk factors for predicting the development of AAD.

**Table 1:** Demographic data of the study population.

Aortic dissection	With (N = 96)	Without (N = 96)	*p* value
**age (years)**	63.4±14.6	63.4±14.5	0.99
**gender (Male)**	76 (78.4%)	76 (78.4%)	1.00
**dissection type (Stanford type A)**	65 (67.0%)	0 (0%)	-
**type 2 diabetes mellitus**	12 (12.5%)	22 (22.9%)	0.09
**hypertension**	81 (84.4%)	34 (35.4%)	<0.01*
**prior cardiac surgery**	4 (4.2%)	5 (5.2%)	0.77
**coronary artery disease**	19 (19.8%)	25 (26.0%)	0.39
**hyperlipidemia**	10 (10.4%)	12 (12.5%)	0.82
**chronic kidney disease**	15 (15.6%)	10 (10.4%)	0.39
**smoking history**	37 (38.5%)	27 (28.1%)	0.17
**presence of AC**	68(70.8%)	67(69.8%)	0.88
**cumulative AC volume**			
ascending aorta	228.0±949.8	63.4±165.7	0.09
aortic arch	211.6±414.5	118.0±183.4	0.04*
descending aorta	395.3±966.0	359.8±763.1	0.78
whole aorta	834.9±1826.6	541.2±1044.5	0.18
**cumulative AC scores**			
ascending aorta	1247.7±3210.2	571.2±1212.7	0.05*
aortic arch	1950.5±2742.5	1161.9±1486.3	0.02*
descending aorta	5634.9±10593.2	5557.1±911.4	0.96
whole aorta	8833.1±15260.9	7290.2±10747.1	0.43

AC: aortic calcification;

* *p* <0.05

**Table 2:** Univariate and multivariate logistic regression analyses regarding the development of acute aortic dissection.

	Univariate			Multivariate	
	HR (95% CI)	*p* value		HR (95% CI)	*p* value
**age**	1.013(0.993–1.033)	0.20	**age**	1.000(0.973–1.028)	0.989
**gender (male)**	1.437(0.689–2.997)	0.33	**gender (male)**	1.672(0.662–4.226)	0.28
**hypertension**	9.655(4.811–19.373)	<0.01*	**hypertension**	9.386(4.883–19.654)	<0.01*
**type 2 Diabetes Mellitus**	1.822(0.829–4.006)	0.14	**aortic calcification**	1.451(0.636–3.308)	0.38
**hyperlipidemia**	1.811(0.775–4.228)	0.17	**coronary artery disease**	3.486(1.340–9.068)	0.01*
**aortic calcification**	1.045(0.555–1.967)	0.89			
**coronary artery disease**	2.351(0.997–5546)	0.05			
**chronic kidney disease**	0.586(0.243–1.413)	0.23			
**smoking history**	0.772(0.424–1.405)	0.40			
**prior cardiac surgery**	1.322(0.344–5.084)	0.69			
**AC scores (10**^**3**^**)**	1.009(0.987–1.032)	0.43			
**AC volume (10**^**2**^ **mm**^**3**^**)**	1.015(0.993–1.037)	0.20			

AC: aortic calcification;

* *p* <0.05

As shown in Table 3, 28 patients (28.9%) with AAD did not have ACs. Compared with AAD patients without ACs, patients with AAD and AC were older, were more frequently male and more frequently had a history of CAD. No significant difference was identified between type A AAD and type B AAD patients without ACs.

**Table 3:** Comparison between acute aortic dissection patients with or without aortic calcification.

Aortic calcification	With (N = 68)	Without (N = 28)	*p* value
**dissection type (type A %)**	48 (70.6%)	16 (57.1%)	0.20
**age (years)**	63.0±13.6	51.6±9.7	<0.01*
**gender (male %)**	48 (70.6%)	27 (96.4%)	<0.01*
**hypertension**	58 (85.3%)	23 (82.1%)	0.70
**type 2 diabetes mellitus**	9 (13.2%)	3 (10.7%)	0.73
**hyperlipidemia**	8 (12.8%)	2 (7.1%)	0.50
**coronary artery disease**	9 (13.2%)	0 (0%)	0.04*
**chronic kidney disease**	9 (13.2%)	6 (21.4%)	0.32
**prior cardiac surgery**	4 (5.9%)	0 (0%)	0.19
**smoking history**	25 (36.8%)	12 (42.9%)	0.58

* *p* <0.05

Among these 64 type A AAD patients, aortic root was the most frequent position of the intimal tear in the ascending aorta (40 are located in aortic root; 16 are located sinotubular junction; 3 proximal, and 5 are in distal ascending aorta). As shown in Table 4, approximately half of the ITs were superimposed on calcified plaques in patients with both types A and B AAD (41.7% and 50.0%, respectively). However, the nearest AC in patients with type A AAD was farther from the IT than that in patients with type B AAD, and it was located distal to the IT. Compared with patients with type B AAD, patients with type A AAD had statistically greater mean AC volumes in the aortic arch, greater cumulative AC volumes in the aortic arch and whole aorta, and higher cumulative AC scores in the aortic arch. We further classified the patients whose initial IT were located at aortic arch into Ishimaru zone classification. Zone 3 (from the distal to the origin of left subclavian artery to an imaginary border at the end of the curvature of aortic arch) was the most frequent site of ITs (42.8%). More than half of the ITs were superimposed on calcified plaques regardless of which Ishimaru classification is (Table 5).

**Table 4:** Comparison between patients with type A and type B acute aortic dissection.

Aortic dissection	Type A (N = 64)	Type B (N = 32)	*p* value
**AC (%)**	48 (75.0%)	20 (62.5%)	0.20
**Mean distance between IT and nearest AC (mm)**	43.0±53.4	10.0±14.0	<0.01*
**Nearest AC location**			<0.01*
Proximal to IT	6 (12.5%)	8 (40.0%)	
Superimposed on IT	20 (41.7%)	10 (50.0%)	
Distal to IT	22 (45.8%)	2 (10.0%)	
**Mean AC volume (mm**^**3**^**)**			
Ascending aorta	38.2±216.0	6.1±11.1	0.40
Aortic arch	15.1±21.3	8.4±9.3	0.04*
Descending aorta	10.6±17.0	6.8±8.0	0.23
Whole aorta	14.6±29.6	6.9±9.7	0.28
**Cumulative AC volume (mm3)**			
Ascending aorta	282.0±1141.2	119.9±315.8	0.43
Aortic arch	274.3±487.2	86.2±141.3	<0.01*
Descending aorta	483.3±1146.8	219.4±372.9	0.21
Whole aorta	1039.6±2163.0	425.5±676.0	0.04*
**Mean AC scores**			
Ascending aorta	98.3±106.8	74.7±107.7	0.31
Aortic arch	146.5±118.5	126.3±108.8	0.42
Descending aorta	170.6±218.6	117.3±113.6	0.20
Whole aorta	150.8±259.6	91.9±133.2	0.14
**Cumulative AC scores**			
Ascending aorta	1282.9±3453.3	1177.2±2708.7	0.88
Aortic arch	2319.1±3079.7	1213.3±1744.8	0.03*
Descending aorta	6559.7±12272.8	3785.3±5700.1	0.23
Whole aorta	10161.7±17497.6	6175.8±8976.0	0.23

IT: intimal tear. AC: aortic calcification.

* *P <0*.*05*

**Table 5:** The associations between the locations of aortic calcification and the aortic arch zones by the Ishimaru classification.

Ishimaru classification	Type 0 (N = 4)	Type 1 (N = 3)	Type 2 (N = 8)	Type 3 (N = 12)	Type 4 (N = 1)
**AC (%)**	4 (100%)	2 (66.7%)	6 (75%)	5 (41.7%)	1 (100.0%)
**Nearest AC location**					
Proximal to IT	1 (25%)	0 (0%)	2 (33.3%)	1 (20%)	0 (0%)
Superimposed on IT	2 (50%)	2 (100%)	3 (50%)	4 (80%)	1 (100%)
Distal to IT	1 (25%)	0 (0%)	1 (16.7%)	0 (0%)	0 (0%)

IT: intimal tear. AC: aortic calcification

### Association of the distance between the IT and the culprit calcified plaque

**Fig 2:**
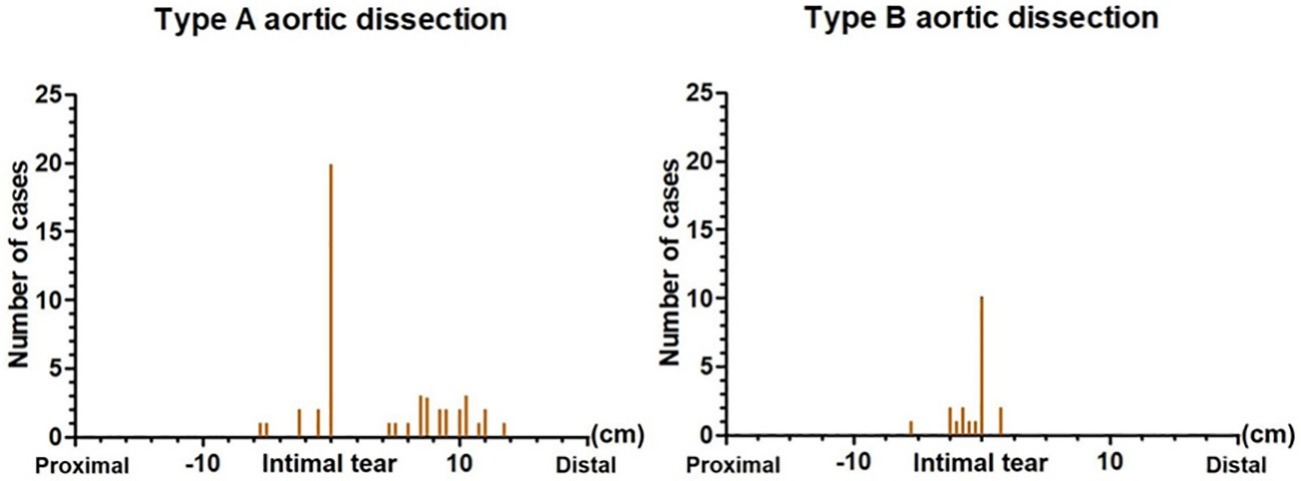
Distribution of aortic calcification and intimal tears in patients with acute aortic dissection.

Regarding patients with type A AAD, as shown in Fig 2, calcified plaques were superimposed on ITs in 20 patients (41.7%) and the nearest calcified plaque was distal to the ITs in the other 22 patients (45.8%). In addition, the nearest calcified plaque was found within 12 cm of the ITs in most cases. By contrast, in patients with type B AAD, calcified plaques were superimposed on ITs in 10 patients (50%), and the nearest calcified plaque was proximal to the ITs in 8 patients (40%). The nearest calcified plaque was found within 2.5 cm of the ITs in most cases.

Regarding the cumulative AC scores, the Kappa value was 0.62 for interobserver reliability, and intraclass correlation coefficient from 0.74 to 0.90. Regarding the distance from the IT, the Kappa value was 0.64 for interobserver reliability, and intraclass correlation coefficient from 0.78 to 0.90. Z-C T and C-J Y respectively read the scans and the intra-observer time differences between the reads was one week.

## Discussion

In the present study, we demonstrated the following: 1) compared with patients without AAD, patients with AAD had a significantly greater AC volume in the aortic arch and significantly greater AC scores for both the ascending aorta and the aortic arch; HTN and CAD, rather than AC, remained to be the independent risk factor for AAD; 2) ACs were superimposed on ITs in nearly half of the patients with AAD; 3) patients with type A AAD had a statistically greater mean AC volume in the aortic arch, greater cumulative AC volumes in the aortic arch and whole aorta and higher cumulative AC scores in the aortic arch than did patients with type B AAD; 4) the nearest AC was located distal to the IT and farther from the IT in patients with type A AAD than that in patients with type B AAD.

Though multivariate regression analysis showed that AC-variables are not crucial factors for the development of AAD, we found that certain associations between AAD and AC do exist especially when it comes to the location of ITs and AC. Once AAD develops, the IT frequently initiates from very near or just on the calcified portion of the aorta. Calcification is an intricate and highly regulated biological process.[14] Although evidence regarding the association between AC and AAD is limited, numerous studies have explored the association between calcification and AAA. Segmental aortic stiffening is an early pathomechanism that generates aortic WSS and triggers AAA growth. Monitoring segmental aortic stiffening may help identify patients at risk for AAA.[15] Disruption of intimal calcifications on multi-detector computed tomography (MDCT) has been suggested as one of the signs of instability in AAA.[16] Intraluminal thrombi and calcifications should both be considered in the evaluation of WSS for risk assessment of AAA rupture.[7] Reversing AC has been proposed as a potential therapeutic target for aortic diseases.[17, 18] Both coronary artery calcification (CAC) and aortic arch calcification (AAC) have been demonstrated to be predictors of future cardiovascular events.[19–22] Risk stratification by the presence of AAC provides important information for the management of atherosclerotic disease.[20, 23] An increase in the volume of a calcified plaque in the ascending aorta has also been associated with CAD, atherosclerotic cardiovascular disease, and ischemic stroke; however, an increase in the density of a calcified plaque alone has been inversely associated with CAD and atherosclerotic cardiovascular disease.[24] AAC is also considered a marker of aortic stiffness.[25] Consistent with previous studies, we demonstrated that increases in AAC volume and scores were positively associated with AAD. Patients with ACs were also older and had significantly higher rates of CAD than patients without AC.

In the present study, we demonstrated an association between the locations of ACs and ITs in patients with AAD. ITs are frequently found coincident with regions of maximal pressure of WSS. High WSS can induce IT formation and result in AAD.[10] Reducing WSS has been proposed to minimize the propagation of dissection.[26, 27] High WSS occurs upstream (proximal) and at the stenotic site of a calcified plaque, which may explain why ACs were found superimposed on nearly half of the ITs in patients with either type A or type B AAD. In a computerized model, high tensile stress was found at junctions between tissue types with differing elastic properties in diseased vessel segments, resulting in ITs.[28] Most coronary artery dissections are found adjacent to calcium deposits after balloon angioplasty, suggesting that localized calcium deposits elicit dissection.[29] ACs can frequently be observed on the outermost wall of false lumens in patients with chronic dissection.[30] Recent animal and human studies have revealed the presence of a positive association between AC and pulse wave velocity and have suggested that AC is a crucial component of aortic stiffness. Fragmentation and degradation of elastin are cardinal structural changes in aortic stiffness. We speculate that once calcification develops, the WSS and biomechanical properties at the site of transition between the normal aorta and the calcified aorta are altered, increasing this area’s vulnerability to ITs. Elastin fragmentation precedes AC, and calcium deposits can also result in damage to elastin fibers in smooth muscle cells.[31] [32] Microcalcifications have been shown to co-localize with aortic elastin degradation in the aortas of humans and mice.[31] [32] In addition to the biomechanical effects of AC, inflammation, WSS, oxidative stress, hyperphosphatemia, and elastolysis further serve as stimuli that promote vascular bone morphogenic protein signaling and matrix remodeling.[33]

Whether type A and type B AAD share the same pathophysiology remains controversial. The demographics and histories of patients with AAD from the International Registry of Acute Aortic Dissection demonstrate that patients with type B AAD were older and had a higher prevalence of atherosclerosis.[34] Elastin is one of the main structural components of the media layer of the arterial wall, and mechanical or functional failure of elastin can predispose the aorta to dissection and degeneration with aneurysm formation. Cystic medial necrosis indicates degeneration of medial collagen and elastin fibers by elastolysis and appears to be an essential feature of several hereditary conditions, such as Ehlers-Danlos syndrome and Marfan syndrome.[35] [36] Elastin decreases progressively from the proximal to distal aorta without a corresponding decrease in collagen; therefore, the distal aorta may have poorer compliance and integrity compared to the proximal aorta.[37] However, the incidence of type B AAD is still much lower than that of type A AAD,[38] suggesting that WSS plays a more important role in the pathophysiology of AAD than does elastin deterioration in the media layer of the aorta. In the present study, we revealed that patients with type A AAD have statistically greater mean and cumulative AC volumes in the aortic arch and greater cumulative AC volumes in the whole aorta than do patients with type B AAD. We postulate that high WSS contributes to AAC and further susceptibility to ITs. The differences in the distances and proximal/distal distributions between ITs and ACs in patients with type A and B AAD should be further investigated through biomechanical models.

The development of AAD is a complex process involving multiple mechanism and signaling pathways.

## Limitations

First, we realize that relative small patient numbers in single medical center setting could have potential bias. we assumed that all the dissections were antegrade and that the very proximal part of the IT was the site of the primary entry tear. Retrograde dissections might have occurred. We are aware that the development of AAD is a complex process involving multiple signaling pathways. We did not specifically differentiate whether calcifications were located in the intima or media layer of the aorta. Further studies with more patients that evaluate hemodynamic and biomechanical parameters should be performed to elucidate these associations.

## Conclusion

We found that patients with AAD had a greater AC volume in the aortic arch and greater AC scores for both the ascending aorta and the aortic arch than did patients without AAD. However, AC was not an independent risk factor for AAD. The distribution of AC was different between patients with type A and type B AAD.

## Supporting information

S1 File Representative value of variables in this study. (XLSX)
